# Study of internal heat source, rotation, magnetic field, and initial stress influence on p-waves propagation in a photothermal semiconducting medium

**DOI:** 10.1038/s41598-024-63568-w

**Published:** 2024-06-25

**Authors:** M. M. Rashid, A. M. Abd-Alla, S. M. Abo-Dahab, F. M. Alharbi

**Affiliations:** 1 Mathematics Department, Faculty of Science, Al-Arish University, Al-Arish, Egypt; 2 Mathematics Department, Faculty of Science, Sohag University, Sohag, Egypt; 3 Mathematics Department, Faculty of Science, South Valley University, Qena, 83523 Egypt; 4 Mathematics Department, Faculty of Science, Umm Al-Qura University, Makkah, 24227 Saudi Arabia

**Keywords:** Rotation, Initial stress, Magnetic field, Photothermal, Semiconductors, Internal heat source, p-waves, Biophysics, Materials science, Mathematics and computing

## Abstract

The purpose of the current study is to establish a novel mathematical model in the p-waves in a photothermal semiconducting medium with an internal heat source. The fundamental equations in the context of isotropic and homogeneous medium have been presented. For the solution of the required problem, the normal mode analysis along with the displacement components, stress components and temperature has been utilized. For graphical representation of different physical quantities such as displacement components, stress components and carrier density as well as the temperature distribution. Using MATLAB R2023a software, a parametric analysis is performed, and the resulting data is represented graphically. A comparison is made to show the effect of the new parameters on the phenomenon. A graphic representation of the relationship between rotation, magnetic field, and initial stress in relation to the fluctuations in non-dimensional field quantities is provided, along with an analysis of the findings.

## Introduction

Therefore, it is still impossible to analyze the elastic response of materials with microstructure using the traditional theory of elasticity. Therefore, Eringen^1^ expanded the scope of classical theory in 1966 by inventing the Micropolar theory of elasticity in order to investigate such types of materials. The displacement vector in micropolar elasticity is not the only thing that a micro-rotation vector that takes into account the granular nature of the medium is also employed to illustrate the deformation of the material particles. It is suggested that the granular character be applied to these kinds of materials in situations where the traditional, conventional elasticity is insufficient to understand their behavior. Later, heat and magnetic effects were added to the micropolar theory of elasticity, expanding its application to certain additional generalizations known as. The classical theory of coupled and uncoupled thermoelasticity was played with the parabolic nature of heat conduction equation, which was leading to the infinite velocity of thermal signals. Therefore, in order to remove this disorder, several generalizations were introduced. The first generalization was established by Lord and Shulman^2^ with one relaxation time named as LS theory and another generalization was given by Green and Lindsay^3^ with two relaxation times named as GL theory. In above LS and GL theories, heat conduction equation is of hyperbolic nature and therefore removes the shortcoming of indefinite velocities of thermal signals. Later on, three additional models were introduced by Green and Naghdi^4–6^ (GN theory), namely G–N type I, II, and III. Semiconducting materials serve an important role in modern engineering, and the electrical conductivity of these materials lies between conductors and insulators. One of the most important characteristic of semiconducting materials is that they possess optical properties. Therefore, when they are subjected to sun light or a laser beam, some of the energy will be soaked up whereas, some of the energy will be liberated in the form of heat or thermal energy. This phenomenon is known as the photothermal effect. This effect is often utilized to measure the thermal properties of the materials especially in semiconductor industry. Furthermore, when a laser beam or sunlight is imposed on elastic medium, a free charge carrier emerges on the surface, which creates the Plasma waves. In other words, plasma waves are developed due to the excited electrons which move randomly on the surface of semiconducting material. The photothermal method was first established by Gordon et al.^7^ when an intracavity spherical semiconducting material sample was subjected to a beam of laser. Employing spectroscopy analysis, Kreuzer^8^ investigated photoacoustic waves generated by a laser source of light. The equations of plasmaelastic and thermoelastic waves were investigated by Todorovic^9^ in a semiconducting plate. A thermoelastic interaction was considered by Abo-Dahab and Lotfy^10^ in order to investigate the photothermal effects on a semiconductor structure. The photothermal interactions were investigated by Hobiny and Abbas^11^ in a 2D semiconducting half-space by employing Green and Naghdi theory. The photo-thermal waves were studied by Mabrouk et al.^12^ in a magneto-rotating semiconducting elastic medium by making use of dual-phase-lag model. A novel mathematical model of a rotating elastic semiconducting medium is developed by Lotfy^13^ in the context of photothermal excitation. Another mathematical model was examined by Raddadi et al.^14^ in the microstretch photo-thermoelasticity theory. Lotfy et al.^15^ investigated Thermomechanical response model on a reflection photothermal diffusion waves (RPTD). The p-waves are the first waves that are detected by a seismograph, which they are the fastest seismic waves and can travel through gases, liquids, or solids. Abo-Dahab^16^ discussed in a semiconducting photothermal diffusion medium p-waves reflection with initial stress and magnetic field. Abd-Alla et al.^20^ discussed the effect of magnetic field and voids on Rayleigh waves in a nonlocal thermoelastic half-space. In recent years, researchers have extensively focused on the p-waves propagation in a photothermal semiconducting medium (see for example^21–25^ and several references therein). In recent years, researchers have extensively focused on the p-waves propagation in a photothermal semiconducting medium (see for example^26–39^ and several references therein).

In the current work, a new mathematical model is developed in the p-waves thermoelasticity by using photothermal semiconducting medium. The components of displacements, stresses and carrier density, as well as the temperature distribution are obtained by utilizing the technique of normal mode analysis. A graphic representation of the relationship between internal heat source, magnetic field, rotation and initial stress in relation to the fluctuations in non-dimensional field quantities is provided, along with an analysis of the findings. A comparison is made to show the differences between the current results and previous results obtained.

## Formulation of the problem

A homogeneous, isotropic, elastic semiconducting material in magneto-thermoelasticity is taken into consideration. Also, the origin of a rectangular cartesian coordinate system (*x*, *y*, *z*) is taken at any point on the plane surface of a half-space, *y* = 0. For the 2D problem, the displacement vector $\overline{u}$ and Maxwell equations (governing the electromagnetic field) in the absence of displacement current with assumption that the silicon crystal medium is perfectly electric conductor are given by Wang and Dong^17^.

1a $$\begin{aligned} & \overrightarrow {J} = \underline{\nabla } \times \underline{h} ,\quad \mu_{e} \frac{{\partial \underline{h} }}{\partial t} = - \underline{\nabla } \times \underline{E} ,\quad div\underline{h} = 0, \\ & div\underline{E} = 0,\quad \underline{E} = - \mu_{e} \left( {\frac{{\partial \underline{u} }}{\partial t} \times \underline{H} } \right),\quad \underline{h} = curl\left( {\underline{u} \times \underline{H} } \right). \\ \end{aligned}$$

Taking into consideration that the Silicon crystal thermo-elastic medium is under constant primary magnetic field $H_{0}$ acting on $z$-axis to the Eq. (1) we have

1b $$\underline{u} = \left( {u,0,w} \right),\quad \underline{H} = \left( {0,H_{0} ,0} \right),\quad \underline{h} = \left( {0, - H_{0} \left( {\frac{\partial u}{{\partial x}} + \frac{\partial w}{{\partial z}}} \right),0} \right),$$

1c $$\overrightarrow {J} \, = \left( {H_{0} \left( {\frac{{\partial^{2} u}}{\partial x\partial z} + \frac{{\partial^{2} w}}{{\partial z^{2} }}} \right),0, - H_{0} \left( {\frac{{\partial^{2} u}}{{\partial x^{2} }} + \frac{{\partial^{2} w}}{\partial x\partial z}} \right)} \right).$$

The Lorentz force vector is

1d $$\overrightarrow {F} = \mu_{e} \left( {\overrightarrow {J} \times \overrightarrow {H} } \right).$$

where

2 $$\overrightarrow {H} = \overrightarrow {H}_{0} + \overrightarrow {h} ,\quad \overrightarrow {H}_{0} = \left( {0,H_{0} ,0} \right),\quad F_{x} = \frac{{\partial \tau_{xx} }}{\partial x},\quad F_{y} = \frac{{\tau_{zz} }}{\partial z}.$$

Considering the plane strain state in the $xz$-plane, where the displacement is defined by

3 $$u = u(x,z,t),\quad w = w(x,z,t).$$

The dynamic equations of motion under a magnetic field, and initial compression stress are given by

4 $$\frac{{\partial \sigma_{xx} }}{\partial x} + \frac{{\partial \tau_{xz} }}{\partial z} - \delta_{n} \frac{\partial N}{{\partial x}} + \mu_{e} H_{0}^{2} \left( {\frac{{\partial^{2} u}}{{\partial x^{2} }} + \frac{{\partial^{2} w}}{\partial x\partial z}} \right) - P\frac{{\partial \omega_{xz} }}{\partial z} = \rho \left[ {\frac{{\partial^{2} u}}{{\partial t^{2} }} - \Omega^{2} u + 2\Omega \frac{\partial w}{{\partial t}}} \right],$$

5 $$\frac{{\partial \sigma_{zz} }}{\partial z} + \frac{{\partial \tau_{xz} }}{\partial x} - \delta_{n} \frac{\partial N}{{\partial z}} + \mu_{e} H_{0}^{2} \left( {\frac{{\partial^{2} w}}{{\partial z^{2} }} + \frac{{\partial^{2} u}}{\partial x\partial z}} \right) - P\frac{{\partial \omega_{xz} }}{\partial x} = \rho \left( {\frac{{\partial^{2} w}}{{\partial t^{2} }} - \Omega^{2} w - 2\Omega \frac{\partial u}{{\partial t}}} \right),$$

The heat conduction equation is

6 $$K\nabla^{2} T + \frac{{E_{g} }}{\tau }N - \gamma T_{0} \left( {1 + \tau_{0} \frac{\partial }{\partial t}} \right)\dot{e} = \rho c_{e} \left( {1 + \tau_{0} \frac{\partial }{\partial t}} \right)\dot{T} - \varepsilon \left( {1 + \tau_{0} \frac{\partial }{\partial t}} \right)Q.$$

The plasma wave equation without plasma source is

7 $$D_{E} \nabla^{2} N - \frac{N}{\tau } + kT = \frac{\partial N}{{\partial t}}.$$

The stress–displacement relations with incremental isotropy are given by

8a $$\sigma_{xx} = \left( {\lambda + 2\mu + P} \right)\frac{\partial u}{{\partial x}} + \left( {\lambda + P} \right)\frac{\partial w}{{\partial z}} - \gamma T,$$

8b $$\sigma_{ZZ} = \left( {\lambda + 2\mu } \right)\frac{\partial w}{{\partial z}} + \lambda \frac{\partial u}{{\partial x}} - \gamma T,$$

8c $$\tau_{xz} = \mu \left( {\frac{\partial u}{{\partial z}} + \frac{\partial w}{{\partial x}}} \right),$$

8d $$\omega_{xz} = \frac{1}{2}\left( {\frac{\partial u}{{\partial z}} - \frac{\partial w}{{\partial x}}} \right),$$

8e $$e = \left( {\frac{\partial u}{{\partial x}} + \frac{\partial w}{{\partial z}}} \right).$$

Equations (4) and (5) with the help of Eqs. (8a)–(8d) change to

9 $$\begin{aligned} & \left( {\lambda + 2\mu + P + \mu_{e} H_{0}^{2} } \right)\frac{{\partial^{2} w}}{{\partial z^{2} }} + \left( {\mu + \frac{P}{2}} \right)\frac{{\partial^{2} w}}{{\partial x^{2} }} - \delta_{n} \frac{\partial N}{{\partial z}} - \gamma \frac{\partial T}{{\partial z}} + \left( {\lambda + \mu - \frac{P}{2} + \mu_{e} H_{0}^{2} } \right)\frac{{\partial^{2} u}}{\partial x\partial z} \\ & \quad = \rho \left( {\frac{{\partial^{2} w}}{{\partial t^{2} }} - \Omega^{2} w - 2\Omega \frac{\partial u}{{\partial t}}} \right). \\ \end{aligned}$$

10 $$\begin{aligned} & \left( {\lambda + 2\mu + P + \mu_{e} H_{0}^{2} } \right)\frac{{\partial^{2} w}}{{\partial z^{2} }} + \left( {\mu + \frac{P}{2}} \right)\frac{{\partial^{2} w}}{{\partial x^{2} }} - \delta_{n} \frac{\partial N}{{\partial z}} - \gamma \frac{\partial T}{{\partial z}} + \left( {\lambda + \mu - \frac{P}{2} + \mu_{e} H_{0}^{2} } \right)\frac{{\partial^{2} u}}{\partial x\partial z} \\ & \quad = \rho \left( {\frac{{\partial^{2} w}}{{\partial t^{2} }} - \Omega^{2} w - 2\Omega \frac{\partial u}{{\partial t}}} \right). \\ \end{aligned}$$

where $$\gamma \, = \,(3\lambda + 2\mu )\alpha_{t}$$, $$\delta_{n} \, = \,(3\lambda + 2\mu )d_{n}$$, $$\kappa = \frac{{\partial N_{0} }}{\partial T}\frac{1}{\tau }$$, $$\varepsilon = \frac{1}{{C_{T}^{3} t^{ * 4} }}$$, $$C_{T}^{2} = \frac{\lambda + 2\mu }{\rho }$$ and $$t^{ * } = \frac{K}{{\rho c_{e} C_{T}^{2} }}$$.

The governing equations can be put into a more convenient form by using the following non-dimensional variables:

11 $$\begin{aligned} & \left( {x^{\prime},z^{\prime},u^{\prime},w^{\prime}} \right) = \frac{1}{{C_{T} t^{ * } }}\left( {x,z,u,w} \right),\;\;\left( {t^{\prime},\tau^{\prime}_{0} } \right) = \frac{1}{{t^{ * } }}\left( {t,\tau_{0} } \right),\;\;T^{\prime} = \frac{\gamma }{(\lambda + 2\mu )}T, \\ & N^{\prime} = \frac{{\delta_{n} }}{(\lambda + 2\mu )}N,\;\;\sigma^{\prime}_{ij} = \frac{1}{\mu }\sigma_{ij} ,\;\;Q^{\prime} = \frac{\gamma }{{K(\lambda + 2\mu )C_{T} t^{ * 2} }}Q \\ \end{aligned}$$

By Helmholtz's theorem^30^, the displacement vector $\overrightarrow {u}$ can be written in the form:

11.a $$\overrightarrow {u} = \underline{\nabla } \varphi + \underline{\nabla } \wedge \overrightarrow {\psi } ,$$

where the two functions $\varphi$ and $\overrightarrow {\psi }$ are known in the theory of elasticity, by Lame's potentials representing irrotational and rotational parts of the displacement vector $\overrightarrow {u}$ respectively.

Introducing the displacement potentials $\phi (x,z,t)$ and $\psi (x,z,t)$ which related to displacement components by the relations:

12 $$\begin{aligned} u & = \frac{\partial \varphi }{{\partial x}} + \frac{\partial \psi }{{\partial z}}, \\ w & = \frac{\partial \varphi }{{\partial z}} - \frac{\partial \psi }{{\partial x}}. \\ \end{aligned}$$

where $$\,\,\overrightarrow {\psi } = (0,\,\psi ,\,0)$$

Using Eqs. (8e), (11) and (12), in Eqs. (6)–(10), we obtain (the dashed above variables have been removed for convenience):

13 $$\left[ {\left( {\rho C_{T}^{2} + P + \mu_{e} H_{0}^{2} } \right)\nabla^{2} - \rho C_{T}^{2} \left( {\frac{{\partial^{2} }}{{\partial t^{2} }} - \Omega^{2} t^{ * 2} } \right)} \right]\phi - \frac{{K^{2} }}{{\rho c_{e}^{2} }}(T + N) + 2\rho C_{T}^{2} \Omega t^{ * } \frac{\partial \psi }{{\partial t}} = 0,$$

14 $$\left[ {\left( {\mu - \frac{P}{2}} \right)\nabla^{2} - \rho C_{T}^{2} \left( {\frac{{\partial^{2} }}{{\partial t^{2} }} - \Omega^{2} t^{ * 2} } \right)} \right]\psi - 2\rho C_{T}^{2} \Omega t^{ * } \frac{\partial \phi }{{\partial t}} = 0,$$

15 $$\left[ {\nabla^{2} - \left( {1 + \tau_{0} \frac{\partial }{\partial t}} \right)\frac{\partial }{\partial t}} \right]T - \varepsilon_{1} \left( {1 + \tau_{0} \frac{\partial }{\partial t}} \right)\nabla^{2} \phi^{ \cdot } + \varepsilon_{2} N = - \left( {1 + \tau_{0} \frac{\partial }{\partial t}} \right)Q,$$

16 $$\left[ {\nabla^{2} - \frac{{Kt^{ * } }}{{\rho c_{e} \tau D_{E} }} - \frac{K}{{\rho c_{e} D_{E} }}\frac{\partial }{\partial t}} \right]N + \varepsilon_{3} T = 0.$$

For the stress–tensor components, we have the following expressions:

17 $$\sigma_{xx} = \beta^{2} \frac{\partial u}{{\partial x}} + \left( {\beta^{2} - 2} \right)\frac{\partial w}{{\partial z}} - \beta^{2} T - \beta^{2} N,$$

18 $$\sigma_{yy} = \left( {\beta^{2} - 2} \right)\frac{\partial u}{{\partial x}} + \beta^{2} \frac{\partial w}{{\partial z}} - \beta^{2} T - \beta^{2} N,$$

19 $$\sigma_{zz} = \left( {\beta^{2} - 2} \right)\nabla^{2} \phi - \beta^{2} T - \beta^{2} N,$$

20a $$\tau_{xz} = \frac{\partial u}{{\partial z}} + \frac{\partial w}{{\partial x}},$$

20b $$\tau_{xy} = \tau_{yz} = 0.$$

where $$\varepsilon_{1} = \frac{{\gamma^{2} T_{0} }}{{K(\lambda + 2\mu )t^{ * } }}$$, $$\varepsilon_{2} = \frac{{\alpha_{t} E_{g} t^{ * } }}{{\rho c_{e} \tau d_{n} }}$$, $$\varepsilon_{3} = \frac{{K\kappa t^{ * } }}{{\rho c_{e} }}$$ and $$\beta^{{2}} = \frac{\lambda + 2\mu }{\mu }.$$

## Normal mode analysis

By breaking down the solution of the physical variables in terms of normal modes, normal mode analysis provides an exact solution that does not presume any limitations on the real physical quantities that are present in the governing equations of the problem under consideration. The following form can be used to decompose the physical quantity solutions in terms of normal modes:

21 $$\begin{aligned} & \left[ {\varphi ,\psi ,T,N,\sigma_{ij} } \right]\left( {x,z,t} \right) = \left( {\varphi^{*} ,\psi^{*} ,T^{*} ,N^{*} ,\sigma^{*}_{ij} } \right)(z)e^{(\omega t + iax)} , \\ & Q = Q_{0} e^{(\omega t + iax)} . \\ \end{aligned}$$

where $\omega$ is the complex time constant (frequency), $i$ is the imaginary unit, $i = \sqrt { - 1}$, *a* is the wave number in the *x*-direction and $\phi^{ * } ,T^{ * } ,N^{ * }$ and $\sigma_{ij}^{ * }$ are the amplitudes of the field quantities $\phi ,T,N,\sigma_{ij}$ and $Q_{0}$ is the magnitude of the internal heat source.

Using Eq. (21), Eqs. (13)–(16) become respectively:

22 $$\left[ {b_{1} \left( {D^{2} - b_{2} } \right) + b_{3} \left( {D^{2} - a^{2} } \right)} \right]\phi^{ * } - b_{4} \left( {T^{ * } + N^{ * } } \right) + b_{5} \psi^{ * } = 0,$$

23 $$\left( {D^{2} - m^{2} } \right)\psi^{ * } - b_{7} \phi^{ * } = 0,$$

24 $$b_{8} \left( {D^{2} - a^{2} } \right)\phi^{ * } + \left( {D^{2} - b_{9} } \right)T^{ * } + \varepsilon_{2} N^{ * } = - \omega_{1} Q_{0} ,$$

25 $$\varepsilon_{3} T^{ * } + \left( {D^{2} - b_{10} } \right)N^{ * } = 0$$

where

$$\begin{aligned} & D^{2} = \frac{{d^{2} }}{{dz^{2} }},\;\omega_{1} = 1 + \tau_{0} \omega ,\;b_{1} = \rho C_{T}^{2} ,\;b_{2} = a^{2} + \omega^{2} - \Omega^{2} t^{ * 2} ,\;b_{3} = P + \mu_{e} H_{0}^{2} ,\;b_{4} = \frac{{K^{2} }}{{\rho c_{e}^{2} }},\;b_{5} = 2b_{1} \Omega t^{ * } \omega \\ & b_{6} = \mu - \frac{P}{2},\;b_{7} = \frac{{b_{5} }}{{b_{6} }},\;b_{8} = - \varepsilon_{1} \omega \omega_{1} ,\;b_{9} = a^{2} + \omega \omega_{1} ,\;b_{10} = a^{2} + \alpha ,\;\alpha = \frac{{Kt^{ * } }}{{\rho c_{e} \tau D_{E} }} + \frac{K\omega }{{\rho c_{e} D_{E} }} \\ & m^{2} = a^{2} + \frac{{b_{1} }}{{b_{6} }}\left( {\omega^{2} - \frac{{b_{5}^{2} }}{{4b_{1}^{2} \omega^{2} }}} \right),\;t^{*} = \frac{{b_{5} }}{{2b_{1} \Omega \omega }}. \\ \end{aligned}$$

Eliminating $\phi^{ * } (z)$, $T^{ * } (z)$ and $N^{ * } (z)$ between Eqs. (22)–(25), we get the following sixth order ordinary differential equations satisfied by $\phi^{ * } (z)$, $T^{ * } (z)$ and $N^{ * } (z)$ can be obtained:

26 $$\left( {D^{8} - PD^{6} + QD^{4} - RD^{2} + S} \right)\left( {\phi^{ * } (z),T^{ * } (z),N^{ * } (z)} \right) = - \ell Q_{0}$$

where Eq. (26) can be factorized as

27 $$\left( {D^{2} - \lambda_{1}^{2} } \right)\left( {D^{2} - \lambda_{2}^{2} } \right)\left( {D^{2} - \lambda_{3}^{2} } \right)\left( {D^{2} - \lambda_{4}^{2} } \right)\phi^{ * } (z) = - \ell Q_{0}$$

where $\lambda_{n}^{2}$, (*n* = 1, 2, 3) are the positive solutions of the following characteristic Eq. (26).

The positive roots of the Eq. (26) are given by

28 $$\begin{aligned} \lambda_{1} & = \frac{1}{2\sqrt 3 }\left[ {\sqrt {3P - \sqrt {3e_{1} } - \sqrt 6 \sqrt {e_{2} - e_{3} } } } \right],\quad \lambda_{2} = \frac{1}{2\sqrt 3 }\left[ {\sqrt {3P - \sqrt {3e_{1} } + \sqrt 6 \sqrt {e_{2} - e_{3} } } } \right], \\ \lambda_{3} & = \frac{1}{2\sqrt 3 }\left[ {\sqrt {3P + \sqrt {3e_{1} } - \sqrt 6 \sqrt {e_{2} + e_{3} } } } \right],\quad \lambda_{4} = \frac{1}{2\sqrt 3 }\left[ {\sqrt {3P + \sqrt {3e_{1} } + \sqrt 6 \sqrt {e_{2} + e_{3} } } } \right]. \\ \end{aligned}$$

where

$$\begin{array}{*{20}l} {\Theta = 2Q^{3} - 9PQR + 27R^{2} + 27P^{2} S - 72QS,} \hfill & {\quad \Theta_{1} = Q^{2} - 3PR + 12S,} \hfill \\ {\Theta_{2} = 3Q^{3} - 9Q(PR + 8S) + 27\left( {R^{2} + P^{2} S} \right),} \hfill & {\quad \Theta_{3} = \left( {\Theta + \sqrt { - 4\Theta_{1}^{3} + \Theta_{2}^{2} } } \right)^{\frac{1}{3}} } \hfill \\ {\Theta_{4} = 3\sqrt 3 \left( {P^{3} - 4PQ + 8R} \right),} \hfill & {\quad e_{1} = 3P^{2} - 8Q + 2^{\frac{7}{3}} \frac{{\Theta_{1} }}{{\Theta_{3} }} + 2^{\frac{5}{3}} \Theta_{3} ,} \hfill \\ {e_{2} = 3P^{2} - 8Q - 2^{\frac{4}{3}} \frac{{\Theta_{1} }}{{\Theta_{3} }} - 2^{\frac{2}{3}} \Theta_{3} ,} \hfill & {\quad e_{3} = \frac{{\Theta_{4} }}{{\sqrt {e_{1} } }}.} \hfill \\ \end{array}$$

$$\begin{aligned} P & = \frac{{G_{2} }}{{G_{1} }},\;\;Q = \frac{{G_{3} }}{{G_{1} }},\;\;R = \frac{{G_{4} }}{{G_{1} }},\;\;S = \frac{{G_{5} }}{{G_{1} }},\;\;\ell = \frac{{G_{6} }}{{G_{1} }},\;\;G_{1} = b_{1} + b_{3} , \\ G_{2} & = - b_{4} b_{8} + b_{1} \left( {m^{2} + b_{2} } \right) + b_{3} \left( {m^{2} + a^{2} } \right) + \left( {b_{9} + b_{10} } \right)\left( {b_{1} + b_{3} } \right), \\ G_{3} & = m^{2} \left( {b_{1} b_{2} + a^{2} b_{3} } \right) + b_{5} b_{7} + \left( {b_{9} b_{10} - \varepsilon_{2} \varepsilon_{3} } \right)\left( {b_{1} + b_{3} } \right) - \left( {b_{9} + b_{10} } \right)\left[ { - b_{1} \left( {m^{2} + b_{2} } \right) - b_{3} \left( {m^{2} + a^{2} } \right)} \right] \\ & \quad - b_{4} b_{8} \left( {m^{2} + a^{2} + b_{10} + \varepsilon_{3} } \right), \\ G_{4} & = - \left( {b_{9} b_{10} - \varepsilon_{2} \varepsilon_{3} } \right)\left( { - b_{1} \left( {m^{2} + b_{2} } \right) - b_{3} \left( {m^{2} + a^{2} } \right)} \right) + \left( {b_{9} + b_{10} } \right)\left[ {m^{2} \left( {b_{1} b_{2} + a^{2} b_{3} } \right) + b_{5} b_{7} } \right] \\ & \quad - b_{4} b_{8} \left( {m^{2} a^{2} + \left( {m^{2} + a^{2} } \right)\left( {b_{10} + \varepsilon_{3} } \right)} \right), \\ G_{5} & = \left( {b_{9} b_{10} - \varepsilon_{2} \varepsilon_{3} } \right)\left( {m^{2} \left( {b_{1} b_{2} + a^{2} b_{3} } \right) + b_{5} b_{7} } \right) - b_{4} b_{8} m^{2} a^{2} \left( {b_{10} + \varepsilon_{3} } \right), \\ G_{6} & = b_{4} \omega_{1} m^{2} \left( {b_{10} + \varepsilon_{3} } \right). \\ \end{aligned}$$

The general solutions of Eqs. (22)–(25), bound as $z \to \infty$, are given by:

29 $$\phi^{ * } (z)\, = \,\sum\limits_{n = 1}^{n = 3} {M_{n} e\,^{{\, - \,\lambda_{n} \,z}} } - r_{0} Q_{0}$$

30 $$\psi^{ * } (z)\, = M_{4} e\,^{\, - \,m\,z}$$

31 $$T^{ * } (z)\, = \,\sum\limits_{n = 1}^{n = 3} {S_{1n} M_{n} e\,^{{\, - \,\lambda_{n} \,z}} } + r_{1} Q_{0}$$

32 $$N^{ * } (z)\, = \,\sum\limits_{n = 1}^{n = 3} {S_{2n} M_{n} e\,^{{\, - \,\lambda_{n} \,z}} } + r_{2} Q_{0}$$

where $M_{n}$, (*n* = 1, 2, 3,4) are constants

$$\begin{aligned} & S_{1n} = \frac{{\left( {\lambda_{n}^{2} - b_{10} } \right)}}{{b_{4} \left[ {\lambda_{n}^{2} - \left( {b_{10} + \varepsilon_{3} } \right)} \right]}}\left[ {b_{1} \left( {\lambda_{n}^{2} - b_{2} } \right) + b_{3} \left( {\lambda_{n}^{2} - a^{2} } \right) + \frac{{b_{5} b_{7} }}{{\lambda_{n}^{2} - m^{2} }}} \right],\;S_{2n} = \frac{{\varepsilon_{3} S_{1n} }}{{b_{10} - \lambda_{n}^{2} }}\;{\text{and}} \\ & r_{0} = \frac{\ell }{E},\;r_{1} = \frac{{r_{0} b_{10} }}{{b_{4} \left( {b_{10} + \varepsilon_{3} } \right)}}\left( {b_{1} b_{2} + a^{2} b_{3} + \frac{{b_{5} b_{7} }}{{m^{2} }}} \right),\;r_{2} = \frac{{r_{1} \varepsilon_{3} }}{{b_{10} }}. \\ \end{aligned}$$

Using Eqs. (12), (21), (29) and (30), the displacement components can be obtained in the following form:

33 $$u^{ * } (z) = \sum\limits_{n = 1}^{n = 3} {iaM_{n} e^{{ - \lambda_{n} z}} } - iar_{0} Q_{0} - mM_{4} e^{ - mz} ,$$

34 $$w^{ * } (z) = - \sum\limits_{n = 1}^{n = 3} {\lambda_{n} M_{n} e^{{ - \lambda_{n} z}} } - iaM_{4} e^{ - mz} .$$

Using Eqs. (17)–(20), (29)–(34),we obtain

35 $$\sigma_{xx}^{ * } (z) = \sum\limits_{n = 1}^{n = 3} {S_{3n} M_{n} e^{{ - \lambda_{n} z}} - 2iam} M_{4} e^{ - mz} + r_{3} Q_{0} ,$$

36 $$\sigma_{yy}^{ * } (z) = \sum\limits_{n = 1}^{n = 3} {S_{4n} M_{n} e^{{ - \lambda_{n} z}} + 2iam} M_{4} e^{ - mz} + r_{4} Q_{0} ,$$

37 $$\sigma_{zz}^{ * } (z) = \sum\limits_{n = 1}^{n = 3} {S_{5n} M_{n} e^{{ - \lambda_{n} z}} } + r_{4} Q_{0} ,$$

38 $$\tau_{xz}^{ * } (z) = - \sum\limits_{n = 1}^{n = 3} {S_{6n} M_{n} e^{{ - \lambda_{n} z}} } + \left( {m^{2} + a^{2} } \right)M_{4} e^{ - mz} ,$$

where

$$\begin{aligned} & S_{3n} = \left( {\beta^{2} - 2} \right)\lambda_{n}^{2} - \beta^{2} a^{2} - \beta^{2} \left( {S_{1n} + S_{2n} } \right),\;S_{4n} = \beta^{2} \left( {\lambda_{n}^{2} - S_{1n} - S_{2n} } \right) - \left( {\beta^{2} - 2} \right)a^{2} , \\ & S_{5n} = \left( {\beta^{2} - 2} \right)\left( {\lambda_{n}^{2} - a^{2} } \right) - \beta^{2} \left( {S_{1n} + S_{2n} } \right),\;S_{6n} = 2ia\lambda_{n} , \\ & r_{3} = \beta^{2} \left( {a^{2} r_{0} - r_{1} - r_{2} } \right),\;r_{4} = a^{2} \left( {\beta^{2} - 2} \right)r_{0} - \beta^{2} \left( {r_{1} + r_{2} } \right). \\ \end{aligned}$$

## Boundary conditions

The constants $M_{n}$ (*n* = 1, 2, 3, 4) have to be chosen such that the boundary conditions on $z = 0$, can be expressed as

39 $$\sigma_{xx} = - p_{1}^{*} e^{(\omega t + iax)} ,\,\,\,\,\tau_{xz} = 0,\,\,\,\,\,\,T = 0,\,\,\,D_{E} \frac{dN}{{dz}} = sN.$$

where $p^{ * }_{1} \,$ and $s$ are constants.

Applying the boundary conditions (39) on the surface $z = 0\,$, we obtain a system of four equations, which is given below:

40 $$\left[ {\begin{array}{*{20}c} {S_{31} } & {S_{32} } & {S_{33} } & { - 2iam} \\ {S_{61} } & {S_{62} } & {S_{63} } & { - \left( {m^{2} + a^{2} } \right)} \\ {S_{11} } & {S_{12} } & {S_{13} } & 0 \\ {\left( {\lambda_{1} + s_{1} } \right)S_{21} } & {\left( {\lambda_{2} + s_{1} } \right)S_{22} } & {\left( {\lambda_{3} + s_{1} } \right)S_{23} } & 0 \\ \end{array} } \right]\left[ {\begin{array}{*{20}c} {M_{1} } \\ {M_{2} } \\ {M_{3} } \\ {M_{4} } \\ \end{array} } \right] = \left[ {\begin{array}{*{20}c} { - p^{ * }_{1} \,} \\ 0 \\ 0 \\ 0 \\ \end{array} } \right].$$

We obtain the values of the constants $M_{n}$ (*n* = 1, 2, 3,4) where $s_{1} = \frac{s}{{D_{E} }}$.

The expressions of $M_{n}$, (*n* = 1, 2, 3,4) obtained by solving the system (40), when substituted in Eqs. (31)–(38), provide us the following expressions of field variables

41 $$u(z) = \left[ {ia\left( {M_{1} e^{{ - \lambda_{1} z}} + M_{2} e^{{ - \lambda_{2} z}} + M_{3} e^{{ - \lambda_{3} z}} } \right) - iar_{0} Q_{0} - mM_{4} e^{ - mz} } \right]e^{\omega t + iax} ,$$

42 $$w(z) = - \left[ {\lambda_{1} M_{1} e^{{ - \lambda_{1} z}} + \lambda_{2} M_{2} e^{{ - \lambda_{2} z}} + \lambda_{3} M_{3} e^{{ - \lambda_{3} z}} + iaM_{4} e^{ - mz} } \right]e^{\omega t + iax} ,$$

43 $$T(z) = \left[ {S_{11} M_{1} e^{{ - \lambda_{1} z}} + S_{12} M_{2} e^{{ - \lambda_{2} z}} + S_{13} M_{3} e^{{ - \lambda_{3} z}} + r_{1} Q_{0} } \right]e^{\omega t + iax} ,$$

44 $$N(z) = \left[ {S_{21} M_{1} e^{{ - \lambda_{1} z}} + S_{22} M_{2} e^{{ - \lambda_{2} z}} + S_{23} M_{3} e^{{ - \lambda_{3} z}} + r_{2} Q_{0} } \right]e^{\omega t + iax} ,$$

45 $$\sigma_{xx} (z) = \left[ {S_{31} M_{1} e^{{ - \lambda_{1} z}} + S_{32} M_{2} e^{{ - \lambda_{2} z}} + S_{33} M_{3} e^{{ - \lambda_{3} z}} - 2iamM_{4} e^{ - mz} + r_{3} Q_{0} } \right]e^{\omega t + iax} ,$$

46 $$\sigma_{yy} (z) = \left[ {S_{41} M_{1} e^{{ - \lambda_{1} z}} + S_{42} M_{2} e^{{ - \lambda_{2} z}} + S_{43} M_{3} e^{{ - \lambda_{3} z}} + 2iamM_{4} e^{ - mz} + r_{4} Q_{0} } \right]e^{\omega t + iax} ,$$

47 $$\sigma_{zz} (z) = \left[ {S_{51} M_{1} e^{{ - \lambda_{1} z}} + S_{52} M_{2} e^{{ - \lambda_{2} z}} + S_{53} M_{3} e^{{ - \lambda_{3} z}} + r_{4} Q_{0} } \right]e^{\omega t + iax} ,$$

48 $$\tau_{xz} (z) = \left[ { - \left( {S_{61} M_{1} e^{{ - \lambda_{1} z}} + S_{62} M_{2} e^{{ - \lambda_{2} z}} + S_{63} M_{3} e^{{ - \lambda_{3} z}} } \right) + \left( {m^{2} + a^{2} } \right)M_{4} e^{ - mz} } \right]e^{\omega t + iax} ,$$

where

$$\begin{aligned} & M_{1} = \frac{{\Delta_{1} }}{\Delta },\quad M_{2} = \frac{{\Delta_{2} }}{\Delta },\quad M_{3} = \frac{{\Delta_{3} }}{\Delta },\quad M_{4} = \frac{{\Delta_{4} }}{\Delta }, \\ & \Delta = S_{31} d_{1} - S_{32} d_{2} + S_{33} d_{3} + 2iamd_{4} , \\ & \Delta_{1} = - p^{ * }_{1} d_{1} ,\;\Delta_{2} = p^{ * }_{1} d_{2} ,\;\Delta_{3} = - p^{ * }_{1} d_{3} ,\;\Delta_{4} = p^{ * }_{1} d_{4} , \\ & d_{1} = \left( {m^{2} + a^{2} } \right)\left[ {S_{13} S_{22} \left( {\lambda_{2} + s_{1} } \right) - S_{12} S_{23} \left( {\lambda_{3} + s_{1} } \right)} \right], \\ & d_{2} = \left( {m^{2} + a^{2} } \right)\left[ {S_{13} S_{21} \left( {\lambda_{1} + s_{1} } \right) - S_{11} S_{23} \left( {\lambda_{3} + s_{1} } \right)} \right], \\ & d_{3} = \left( {m^{2} + a^{2} } \right)\left[ {S_{12} S_{21} \left( {\lambda_{1} + s_{1} } \right) - S_{11} S_{22} \left( {\lambda_{2} + s_{1} } \right)} \right], \\ & d_{4} = S_{61} \left[ {S_{12} S_{23} \left( {\lambda_{3} + s_{1} } \right) - S_{13} S_{22} \left( {\lambda_{2} + s_{1} } \right)} \right] - S_{62} \left[ {S_{11} S_{23} \left( {\lambda_{3} + s_{1} } \right) - S_{13} S_{21} \left( {\lambda_{1} + s_{1} } \right)} \right] \\ & \quad \quad + S_{63} \left[ {S_{11} S_{22} \left( {\lambda_{2} + s_{1} } \right) - S_{12} S_{21} \left( {\lambda_{1} + s_{1} } \right)} \right] \\ \end{aligned}$$

## Numerical results and discussion

In order to illustrate the theoretical results obtained in the preceding sections, we now present some numerical results. The numerical work has been carried out with the help of computer programming using the software MATLAB R2023a. The behavior of the field quantities is shown in relation to the characteristic time of the pulse heat flux, $t_{p}$. The silicon crystal (Si) materiel is chosen to represent and semiconductor elastic material. The physical constant of Si is used to analyze the results of the phenomenon numerically and discussed. The physical constants in SI unit of silicon crystal (Si) are given below as^18,19^ (Table 1).

**Table 1:** Physical constants of the chosen material^18,19^.

$\lambda { = 3}{\text{.64}} \times {10}^{{{10}}}$ Nm^−2^	$\mu = {5}{\text{.46}} \times {10}^{{{10}}}$ kg m^−1^ s^−2^	$K = 150$ W m^−1^ K^−1^	$\alpha_{t} = 3 \, \times 10^{ - 6}$ K^−1^	$x = 0.05$
$s = 2$	$\kappa = 8.3 \times {10}^{9}$	$\rho = 2.33 \times 10^{3}$ kg m^−3^	$C_{E} = 695$ J kg^−1^ K^−1^	$T_{0} = 800$ K
$a{ = }1.5$	$p_{1}^{ * } { = 0}.1$	*τ*_0_ = 0.02	*t* = 0.01	$\omega = \omega_{0} + i\zeta$
$\omega_{0} = 5$,$\tau = 5 \times {10}^{{ - 5}}$	$\zeta = 0.01$	$d_{n} = - 9 \times {10}^{{ - 31}}$	$D_{E} = 2.5 \times {10}^{{ - 3}}$	$E_{g} = 1.12$

**Figure 1:**
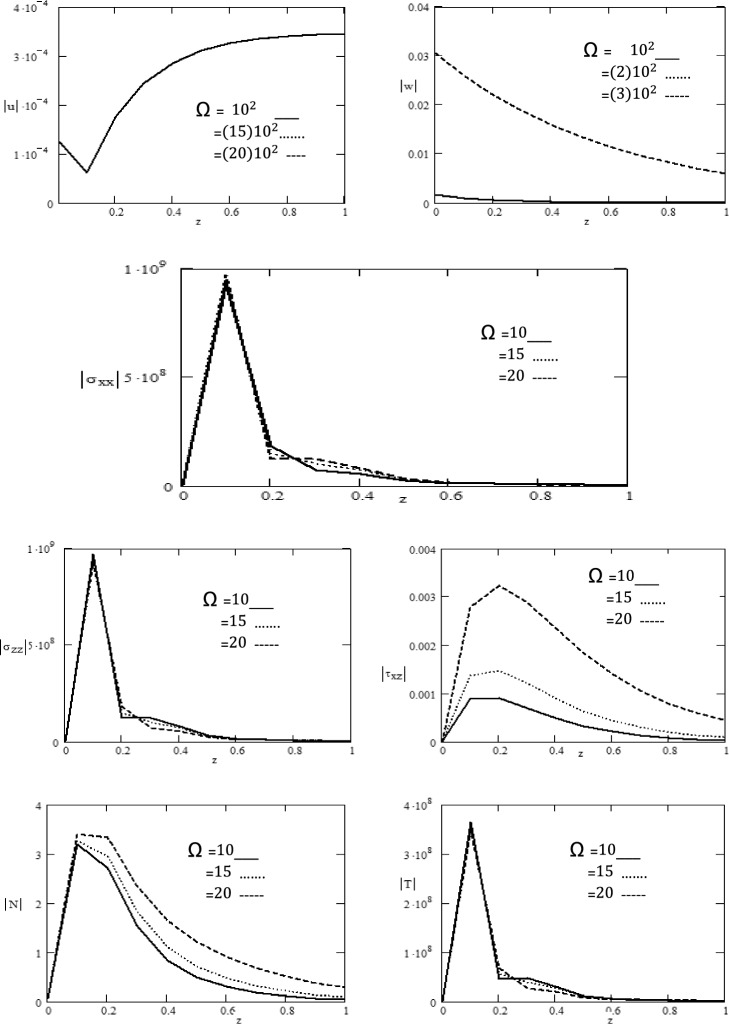
Variations of magnitude of $u,\,w,\,\sigma_{xx} ,\,\sigma_{zz} ,\tau_{xz} ,\,N,\,T$ for different values of rotation $\Omega$ with respect to distance $z$.

**Figure 2:**
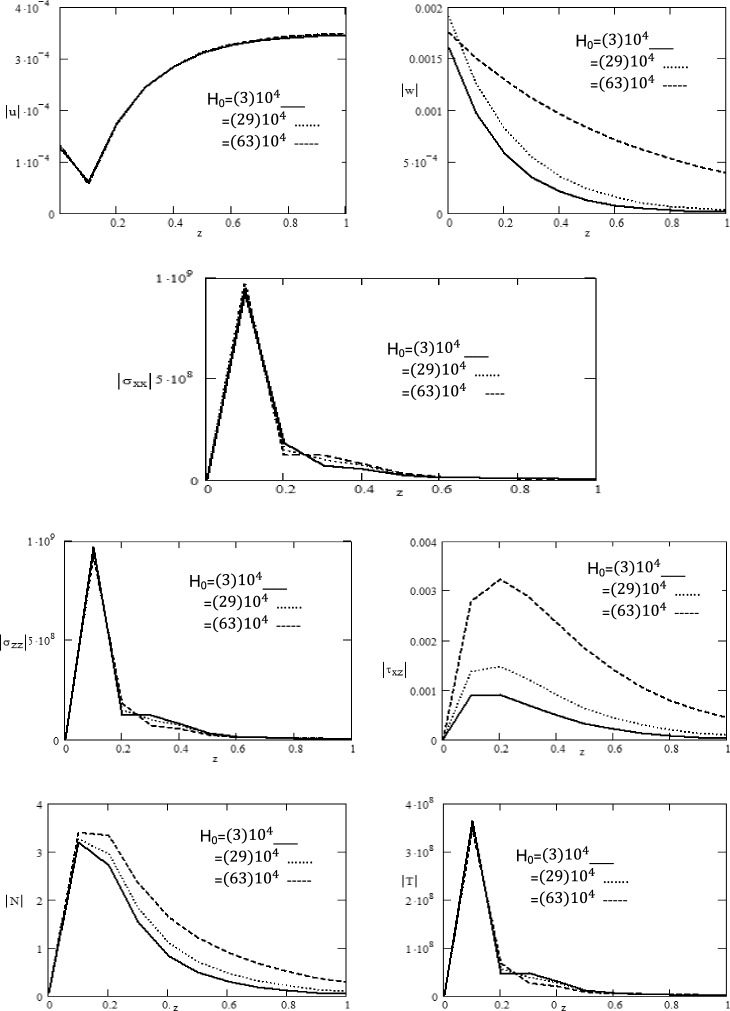
Variations of magnitude of $u,\,w,\,\sigma_{xx} ,\,\sigma_{zz} ,\tau_{xz} ,\,N,\,T$ for different values of magnetic field $H_{0}$ with respect to distance $z$.

**Figure 3:**
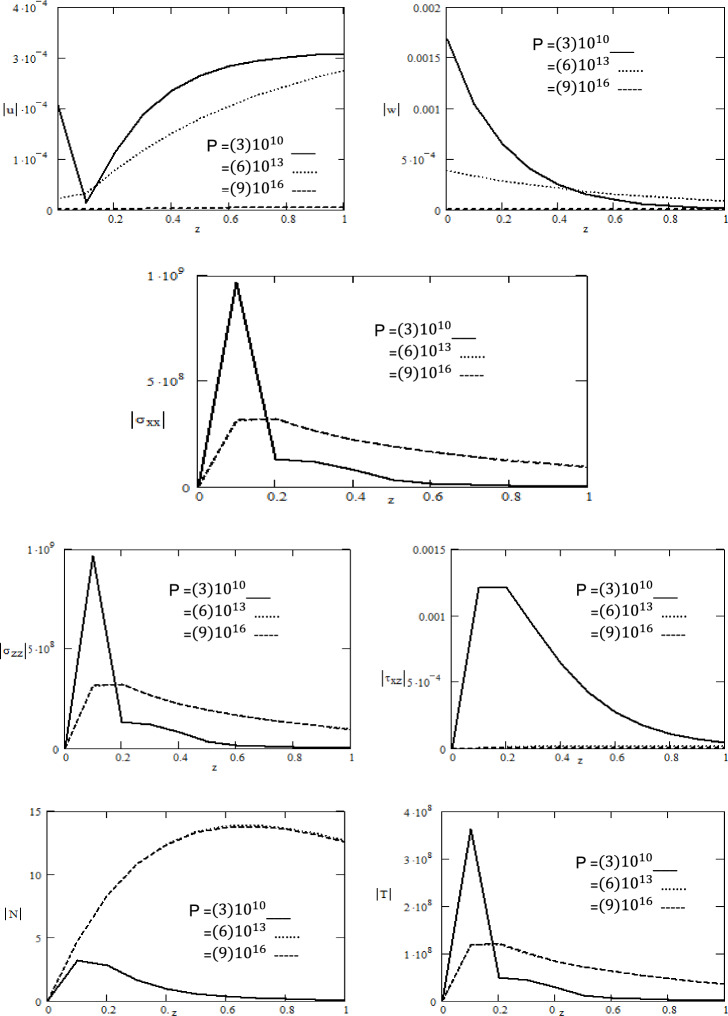
Variations of magnitude of $u,\,w,\,\sigma_{xx} ,\,\sigma_{zz} ,N,\,T$ for different values of initial stress $P$ with respect to distance $z$.

Using the previously mentioned normal mode analysis, the components of displacement, stresses, temperature distribution, and carrier density are obtained. At different $z$ positions, the results graphically shown for magnetic field different values $H_{0}$, initial stress $p$ and rotation $\Omega$ as shown in Figs. 1, 2 and 3.

Figure 1 displays the rotation effect $\Omega$ on the absolute values for components of displacement $\left| u \right|,\,\left| w \right|,$ normal stress components $\left| {\sigma_{xx} } \right|,\,\left| {\sigma_{zz} } \right|$, absolute value of tangential stress $\left| {\tau_{xz} } \right|,$ absolute value of carrier density $\left| N \right|$ and absolute value of temperature $\left| T \right|$ with respect to distance $z$. Figure 1 clearly reveals that the components of displacement $\left| u \right|$ exhibits decreases and increases in the region 0 ≤ $z$ ≤ 1 and there is no influenced by $\Omega ,$ while the components of displacement $\left| w \right|$ decreases in the region 0 ≤ $z$ ≤ 1 and is greatly influenced by $\Omega$. It is obvious from the graph that the components of normal stress $\left| {\sigma_{xx} } \right|,\,\left| {\sigma_{zz} } \right|$ and temperature $\left| T \right|$ keep increasing, decreasing and there is no effect of rotation in the range 0 ≤ $z$ ≤ 0.2, while it exhibits an oscillatory behavior in the region 0.2 ≤ $z$ ≤ 1 and is greatly influenced by $\Omega ,$ as well tangential stress $\left| {\tau_{xz} } \right|,$ and carrier density $\left| N \right|$ increase with the rotation increasing $\Omega$ and it satisfied the boundary conditions. From the graph, it is clearly observed $\left| N \right|$ shows same behavior as that of $\left| T \right|$ in terms of amplitude. Additionally, it is evident that the amplitude of the components of displacement $w$ is highest for $\Omega = 3 \times 10^{2} .$ The intriguing behavior seen may be due to the intricate characteristics of temperature and other physical quantities, this is well in agreement with the physical situation and consistent with the results obtained by^26,27^. Figure 2 demonstrates the magnetic field effect $H_{0}$ on the absolute values of displacement components $\left| u \right|,\,\left| w \right|,$ absolute value of components of normal stress $\left| {\sigma_{xx} } \right|,\,\left| {\sigma_{zz} } \right|$, absolute value of tangential stress $\left| {\tau_{xz} } \right|,$ absolute value of carrier density $\left| N \right|$ and absolute value of temperature $\left| T \right|$ with respect to distance $z$. We observe that the components of displacement $u$ exhibits decreases and increases in the region 0 ≤ $z$ ≤ 1 and there is no influenced by $H_{0} ,$ while the absolute value of displacement component $\left| w \right|$ decreases in the region 0 ≤ $z$ ≤ 1 and is greatly influenced by $H_{0} .$ It is obvious from the graph that the absolute value of normal stress components $\left| {\sigma_{xx} } \right|,\,\left| {\sigma_{zz} } \right|$ and absolute value of temperature $\left| T \right|$ keep increasing, decreasing and there is no effect of magnetic field in the range 0 ≤ $z$ ≤ 0.2, while it exhibits an oscillatory behavior in the region 0.2 ≤ $z$ ≤ 1 and is greatly influenced by $H_{0} ,$ as well absolute value of tangential stress $\left| {\tau_{xz} } \right|$ and absolute value of carrier density $\left| N \right|$ increase with increasing the magnetic field $H_{0}$ and it satisfied the boundary conditions. Additionally, it is evident that the amplitude of the displacement component $w$ is highest for $H_{0} = 63x10^{6} .$ The one common observation in $\left| w \right|,\,\left| {\tau_{xz} } \right|$ and $\left| N \right|$ is higher the value of $H_{0}$, higher is the amplitude. This is mainly due to the fact that effect of magnetic field corresponds to the term signifying positive forces that tend to accelerate the metal particles. This behavior harmoniously aligns with the anticipated traits of normal stress and shear stress within the semiconductor medium described by Ailawalia^28^.

In Fig. 3, attempts have been made to explore the effects of initial stress $P$ on the variation in the absolute values of displacement components $\left| u \right|,\,\left| w \right|,$ absolute value of normal stress components $\left| {\sigma_{xx} } \right|,\,\left| {\sigma_{zz} } \right|$, absolute value of tangential stress $\left| {\tau_{xz} } \right|$, absolute value of carrier density $\left| N \right|$ and absolute value of temperature $\left| T \right|$ with respect to distance $z$. Figure 3 clearly reveals that the displacement components $u,\,w$ decrease with increasing initial stress in the region 0 ≤ $z$ ≤ 1 and is greatly influenced by $P.$ It is obvious from the graph that the normal stress components $\left| {\sigma_{xx} } \right|,\,\left| {\sigma_{zz} } \right|$ and temperature $\left| T \right|$ keep decreasing with increasing $P$ in the range 0 ≤ $z$ ≤ 0.2,while it increases with increasing of $P$ in the region 0.2 ≤ $z$ ≤ 1 and is greatly influenced by $p$ as well tangential stress $\tau_{xz}$ decreases and carrier density $N$ increase with increasing the initial stress $P$ and it satisfied the boundary conditions. Additionally, it is evident that the amplitude of the displacement component $w$ is highest for $P = 3x10^{10} .$ The behavior of normal stress $\left| {\sigma_{xx} } \right|$ is identical as observed in the case of normal stress $\left| {\sigma_{zz} } \right|$. This behavior seamlessly aligns with the expected characteristics of displacement components within semiconductor mediums, Lotfy et al.^29^. The stress curves are showing singularity due to coupling of thermoelastic field between internal heat source, rotation, initial stress and magnetic fields.

## Conclusion

The main goal of the present work is introduced a new model which describe the internal heat source, rotation, magnetic field, and initial stress effect of semiconductor thermoelastic medium in context of photothermal excitation. In the current investigation, homogeneous, isotropic, semiconducting material (silicon) has been considered in the current inquiry along with a newly proposed mathematical model appropriate for the thermoelastic media that also takes the photothermal effect into account. We can conclude that the rotation, magnetic field and initial stress have a significant effect on the absolute of displacement components, normal stresses, tangential stress, carrier density and temperature during photothermal transfer process. A numerical and graphical discussion of the study's findings indicates that the magnetic field, rotation, and initial stress all have a significant impact on various physical variables. If the new parameter is ignored, the produced findings are consistent with the earlier estimation. Normal mode analysis has been used to mathematically solve the problem. That can be used for a variety of thermoelasticity-related issues. The investigation of the initial stress, rotation, and magnetic field in a photo-thermoelastic media can be mainly.

## Data Availability

The datasets used and/or analyzed during the current study available from the corresponding author on reasonable request.

## List of symbols

$$t$$: The time
$T_{0}$: The absolute temperature
$\overrightarrow {u}$: The displacement vector
$\rho$: The material density
${c}_{e}$: The specific heating at constant strain
*N*: The carrier density
$\lambda , \mu$: The Lame’s constants
$\mu_{e}$: The magnetic permeability
$H_{0}$: The primary magnetic field
$\overrightarrow {h}$: The induced magnetic field vector
$\overrightarrow {E}$: The induced electric field vector
$\overrightarrow {J}$: The current density vector
$P$: The initial stress
$\Omega$: The angular velocity
${D}_{E}$: The coefficient of carrier diffusions
$\tau_{0}$: The thermal relaxation time
$\tau$: The photo-generated carrier lifetime
$Q$: The power of the source of heat per unit mass
$\tau$: The photogenerated carrier lifetime
$d_{n}$: The coefficient of electronic deformation
$\delta_{n}$: The difference of deformation potential of conduction and valence band
${E}_{g}$: The energy gap
$K$: The thermal conductivity of the sample
$\alpha_{t}$: The thermal expansion coefficient
$N_{0}$: The carrier concentration at temperature
$\overrightarrow {F} ,$: The Lorentz's body force vector
$\sigma_{ii}$ and $\tau_{ij}$: The stresses tensors
$\varepsilon_{ij}$: The strain components
$T$: The temperature above the reference temperature $T_{0}$
$\delta_{ij}$: The Kronecker delta
$E_{g}$: The energy gap of semiconductor
